# Birth defects and their impact on child morbidity and mortality in developing settings

**DOI:** 10.11604/pamj.2024.48.150.44701

**Published:** 2024-08-05

**Authors:** George Nyadimo Agot, Marshal Mutinda Mweu, Joseph Kibuchi Wang'ombe

**Affiliations:** 1 Department of Public and Global Health, Faculty of Health Sciences, University of Nairobi, Nairobi, Kenya

**Keywords:** Birth defects, child morbidity, child mortality, impact

## Abstract

Despite the notable gains that have been realized in reversing perinatal, neonatal, and childhood morbidity and mortality, insignificant actions on birth defects undermine the desired outcomes. A yearly upward trend of birth defects (44.04-205.28 per 100,000 livebirths) between 2014 and 2018 attributed to known genetic, unknown multifactorial inheritance, and socio-demographic environmental factors, with an estimated unit economic cost of $ 1,139.73 for outpatient services was observed in Kiambu County, Kenya. Thus, interventions anchored on social health insurance would suffice.

## Brief

Birth defects are defined as structural or functional congenital malformations occurring during the intrauterine period and detectable prenatally, at birth, or later [1]. Even though major birth defects have been known to contribute significantly to neonatal and childhood morbidity and mortality, the occurrence of minor birth defects cannot be ignored either, owing to their sequential relationship [2]. Thus, underpins the need to understand the public health burden of all forms of birth defects in the region. About 30% to 50%, and 5% to 7% of perinatal (miscarriages and stillbirths), neonatal, and childhood mortality have been attributed to birth defects in developed and developing countries respectively [3]. Even though notable gains have been realized in reversing such poor health outcomes, taking deliberate actions against the rising trends of birth defects would further reduce perinatal, neonatal, and childhood morbidity and mortality worldwide.

Approximately 7.9 million births occurring worldwide annually are birth-defect related with about 3.3 million dying before attaining at least five years of age whilst 3.2 million could be physically disabled for life [1]. More than 94% of these defects are observed in developing settings of which approximately 95% of children affected by such defects do not live beyond childhood [1]. A similar phenomenon was observed in Kiambu County, Kenya noting a steady upward trend of major external structural birth defects ranging between 44.04 to 205.28 per 100,000 live births between 2014 and 2018 [2]. The birth defects observed in the county consisted of those of the musculoskeletal system (57.46%), central nervous system (17.13%), orofacial defects (13.26%), defects of genital organs (11.05%), as well as anal and eye defects at 0.5% each [2]. Identifiable genetic factors proxied by history of siblings with birth defects, socio-demographic-environmental factors pointed to by maternal residence at conception, and unknown multifactorial inheritance factors were noted as the primary predictors of the occurrence of these defects in the county [1,4].

**Public health implications:** as a result of these defects, the county could have experienced a significant opportunity cost on its budget or would have been required to allocate substantial resources for birth defects in the county during the study period spanning between 2014 and 2018 [5-7]. Of the 362 cases observed, approximately 19% (67) of the defects consisting of musculoskeletal and central nervous system defects were potentially fatal and could contribute substantially to perinatal, neonatal, and childhood mortality in the county between 2014 and 2018 in Kiambu County. This implied that for every 100,000 live births, 19 such deaths could have been due to birth defects such as conjoint twins (1), gastroschisis (12), and omphalocele (8) (defects of the musculoskeletal system); and those of the central nervous system comprising anencephaly (19), hydrocephaly (16), microcephaly (4), craniorachischisis (2), meningocele (2), neurological defect (1), sacrococcygeal teratoma (1), and craniosynostosis (1). With an estimated unit economic cost of $ 1,139.73 for each case of outpatient care, this phenomenon could have impacted significantly the county´s resource envelope [2,8]. This would have implied an opportunity cost of approximately $ 412,582.26 (KES 53,635,693.8) traded-off in the county´s health resource envelope between 2014-2018 to outpatient services at an exchange rate of KES 130.00 per US Dollar for major birth defects obvious at birth. On the other hand, the unit economic cost of outpatient care for neural tube defect was estimated at $ 1,143.51, whilst that of clubfoot and congenital pes planus were estimated at $ 1,143.05, and $ 1,109.81, respectively [2,8]. Undoubtedly, these resources would otherwise be applied to improving the social development infrastructure such as health, and education in the county if the prevalence of these defects would be optimally reduced.

**Conclusions and recommendations:** we conclude that major birth defects are still on an upward trajectory, particularly in regions without strategic public health actions against such congenital anomalies. Moreover, the effects of such interventions would be substantially supplemental to the already known low-cost yet high-impact interventions in reversing the trends of perinatal, neonatal, and childhood morbidity and mortality in developing countries. Further, we recommend specific public interventions against major birth defects comprising the establishment of: (1) hospital-based surveillance systems for birth defects, (2) health promotion messages tailored to women of reproductive age, (3) scaling-up treatment strategies for birth defects, and (4) integrating genetic clinical services consisting of counseling, screening, diagnosis, and related treatments with reproductive health services all anchored on social health insurance principles.
